# Visible-infrared compatible and independent camouflage with multicolor patterns and tunable emissivity

**DOI:** 10.1515/nanoph-2024-0125

**Published:** 2024-05-17

**Authors:** Yuetang Wang, Liming Yuan, Yong Mao, Cheng Huang, Jingkai Huang, Xiaoliang Ma, Yuzhuo Qi, Yang Liu, He Lin, Xiangang Luo

**Affiliations:** State Key Laboratory of Optical Technologies on Nano-Fabrication and Micro-Engineering, 74709 Institute of Optics and Electronics, Chinese Academy of Sciences, Chengdu 610209, China; School of Optoelectronic Science and Engineering, University of Electronic Science and Technology of China, Chengdu 610054, China; National Key Laboratory of Optical Field Manipulation Science and Technology, Chinese Academy of Sciences, Chengdu 610209, China; College of Materials Sciences and Opto-Electronic Technology, University of Chinese Academy of Sciences, Beijing 100049, China; The 722 Research Institute of China State Shipbuilding Corporation Limited, Wuhan 430205, China

**Keywords:** visible-infrared camouflage, amorphous photonic structure, multicolor patterns, tunable emissivity, large-area fabrication

## Abstract

With the rapid development and wide application of visible (VIS) and infrared (IR) detections, it is necessary to explore visible-infrared (VIS-IR) compatible camouflage. Here, we report a VIS-IR compatible and independent camouflage device which is composed of the upper IR-transparent VIS-color-patterned layer and the lower electrochromic IR layer. The upper layer has amorphous photonic structure of polystyrene nanospheres (PSNSs). By customizing the PSNS size, various colors can be realized for VIS camouflage. The lower electrochromic IR layer takes advantage of multiwall carbon nanotubes (MWCNTs) as the electrode as well as the IR active material. Experimental results reveal that different colors (including blue, green, and purple) have been obtained, and the IR emissivity can be electrically regulated from 0.43 to 0.9. Moreover, the prototype also exhibits good electrical stability as well as hydrophobic characteristic (the water contact angle of the outmost surface exceeds 120°). These output performances demonstrate the success of our design strategy for promoting the finding applied in camouflage fields as well as energy conservation fields.

## 1 Introduction

In order to response the rapid development of visible (VIS) and infrared (IR) detections, the corresponding VIS-IR compatible camouflage urgently needs to be explored. In visible regime, the camouflage color is mainly divided into pigment color (originated from wavelength-selective absorption by molecules) and structural color (originated from wavelength-selective reflection by periodic fine structures). On one hand, by exploiting organic binder to combine metallic particles (e.g., flaky aluminum powders) and pigment together, various VIS-IR camouflage coatings have been studied [1], [2], [3]. It is noticed that most of colored pigments feature high IR absorption, leading to hardly realizing low IR emissivity. Meanwhile, the kind of camouflage couples the VIS and IR functionalities together, i.e., darkly colored pattern has higher IR emissivity while lightly colored one has lower emissivity, resulting in a great challenge for the camouflage effect under the combined VIS-IR detection. On the other hand, structural color originates from light scattering, diffraction and interference [4], can be implemented by photonic structure including photonic crystals [5], [6], [7], multilayer film [8], [9], [10], [11], [12], [13], [14], [15] and others. Through elaborate design, the structure can present colorful appearance as well as good compatibility with other wavelengths. However, different structural colors require different structures, meaning that it will be difficult to realize large-area multicolor preparation. Hence, high-performance and easy-preparation VIS-IR compatible camouflage still needs to be further explored. Besides, dynamic IR modulators have received extensive attention, which can dynamically produce on-demand IR feature to match with changing backgrounds, especially for motion objects. According to Stefan–Boltzmann law, the radiated IR signal from an object is proportional to surface emissivity and the quadruplicate of surface temperature. Thereby, IR radiation can be regulated through manipulating the temperature [16], [17], [18], [19] or the emissivity [20], [21], [22], [23], [24], [25], [26], [27], [28], [29], [30], [31], [32], [33], [34], [35], [36], [37], [38]. By comparison, the temperature manipulation approach requires complex devices and sufficient time to achieve heating and cooling, while the emissivity one is more feasible. Many IR emissivity regulators, such as WO_3_-based [20], [21], [22], polyaniline (PANI)-based [23], Li_4_Ti_5_O_12_-based (LTO) [24], carbon-based films [25], [26], [27], [28], [29], [30], VO_2_-based [31] and Ge_2_Sb_2_Te_5_-based (GST) [32] have been demonstrated to well realize the dynamical regulation of the IR emissivity. However, shortages still remain in multispectral compatibility and independence. Recently, an all-solid-state electrochromic device with a Ge/HfO_2_ Fabry–Perot cavity structure successfully demonstrated the VIS-IR compatible and decoupled camouflage performance, but its emissivity modulation capability and the difficult-fabricated color-patterned photonic structure need to be further improved [22]. In summary, to better satisfy the requirements of the VIS-IR compatible camouflage, we need to pay more attention to three aspects: (1) easy preparation of large-area multicolor patterns; (2) decoupling of the VIS and IR functionalities; (3) dynamically regulation of VIS or IR features for matching with changing environment.

In this study, we proposed a VIS-IR compatible and independent camouflage device which is composed of the upper IR-transparent VIS-color-patterned layer and the lower electrochromic IR layer. Here, the upper layer has amorphous photonic structure of polystyrene nanospheres (PSNSs). By customizing the PSNS size, various colors can be realized for VIS camouflage. The lower electrochromic IR layer takes advantage of MWCNTs as the electrode as well as the IR active material. Experimental results reveal that different colors have been obtained, and the IR emissivity can be electrically regulated from 0.43 to 0.9. Moreover, the prototype shows good hydrophobic characteristic and electrical stability for practical applications. Hence, it is believed that our device provides an efficient approach to VIS-IR compatible manipulation technology.

## 2 Results and discussion

### 2.1 Design and simulations

**Figure 1:**
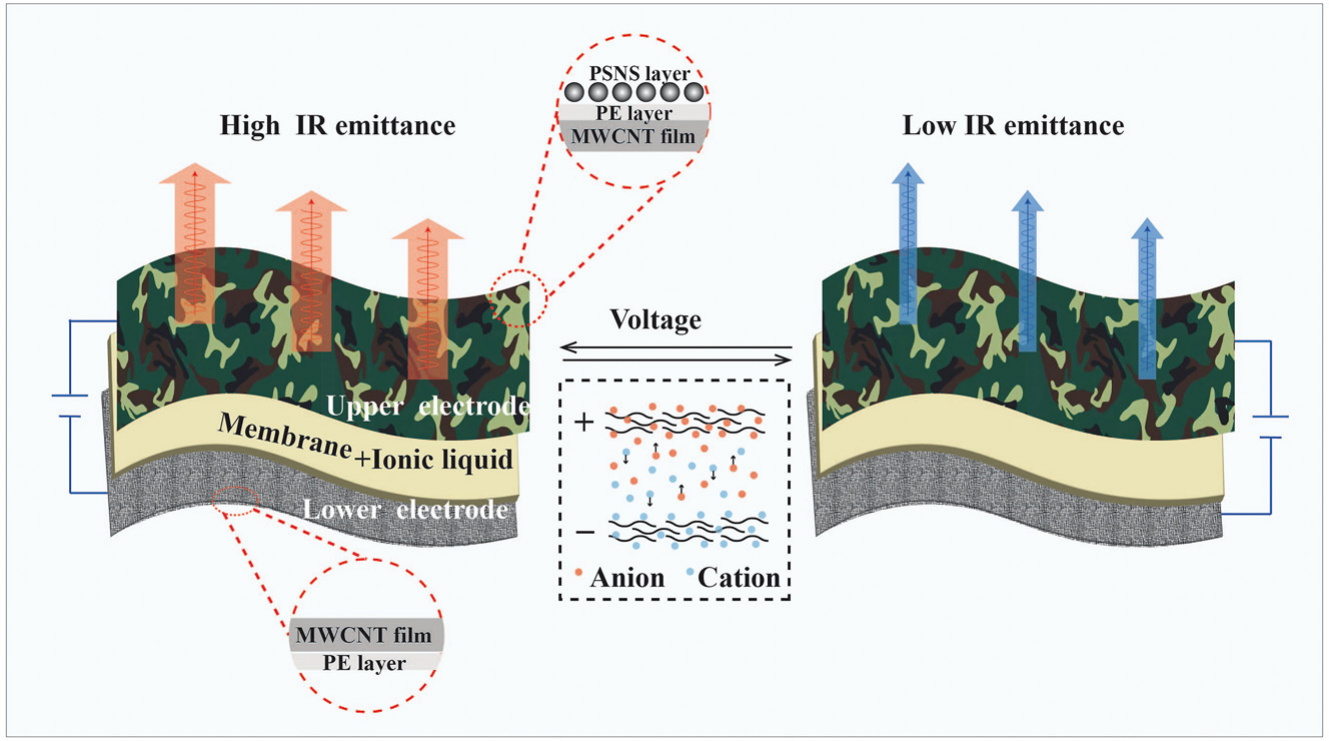
Schematic illustration of our VIS-IR compatible camouflage device. VIS multicolor patterns are realized by amorphous photonic structure of PSNSs and IR emissivity modulation is implemented by charging/discharging MWCNTs.

Figure 1 illustrates the capacitor-like structure of our camouflage device, which is composed of the upper multilayer MWCNT/PE/PSNS composited film, porous PE spacer filled with ionic liquid, and the lower MWCNT/PE composited film. In our design, both the VIS and IR functions come from the upper multilayer film, i.e., the outermost PSNS layer is used to realize customized colors, meanwhile, it also has high IR transparency; and the innermost MWCNT layer is used as active material for IR emissivity regulation as well as an electrode of the whole device. Significantly, the VIS and IR camouflage functions are implemented independently, rather than coupled together as like as most existing VIS-IR compatible camouflages.

In order to realize angle-independent colors, we introduce the short-range ordered amorphous photonic structure (a kind of non-closed-packed face centered-cubic crystal structure) into the PSNS layer. This structural can create a forbidden gap (photonic stopband), and then reflect those forbidden lights to display specified colors. Its stopband position (*λ*) can be calculated according to Bragg diffraction Equation (1) and Equations (2) and (3) [39], [40].

(1) $$m\lambda =2d_{111}\sqrt{n_{\text{eff}}^{2}-sin^{2}\theta }$$

(2) $$d_{111}=\sqrt{2/3}D$$

(3) $$n_{\text{eff}}^{2}=n_{ps}^{2}\varphi_{ps}+n_{\text{matrix}}^{2}\left(1-\varphi_{ps}\right)$$

in which, *d*_111_ is the distance between two neighboring (111) planes, *n*_eff_ is an effective refractive index and *θ* is the angle between incident beam and the normal direction; *D* is the PSNS diameter; *n*_ps_ and *n*_matrix_ are the refractive indexes of the PSNS and the matrix, respectively; *φ*_*ps*_ is the PSNS volume fraction. When *D* = 200 nm, *n*_*ps*_ ≈ 1.6, *n*_matrix_ ≈ 1.0 (air), and *φ*_*ps*_ ∼ 0.74, the stopband position *λ* is calculated as 478 nm. Meanwhile, it is feasible to obtain other stopband positions by just changing the PSNS diameter.

As for IR emissivity modulation, commercial MWCNT film is employed as the active material because of their excellent electrically tunable property in IR regime and easy large-area fabrication for practical application potential. Besides, the high electrical conductivity of the MWCNT film eliminates the requirement for an additional electrode layer. When a positive voltage is applied on the upper MWCNTs electrode, anions will be driven to the MWCNT and then the absorbed anions are intercalated into the graphite layer of MWCNTs, which will lead to the P-type doping. Similarly, the cations will lead to N-type doping under negative voltage. Both p-doping and n-doping can shift Fermi level and suppress the interband transitions, leading to the change of infrared absorptivity as well as emissivity. The intercalation process is reversible by altering the biasing voltage, thus the emissivity of our camouflage device can be dynamically regulated. Subsequently, infrared-transparent PSNSs are utilized to further realize independent VIS functionality. However, PSNSs cannot be directly deposited onto the surface of the MWCNT film, because the ionic liquid will gradually infiltrate into the PSNS layer and replace the air interspace among PSNSs. According to Equation (4), the photon bandgap width (*W*_band_) is proportional to the difference of the refractive index between the PSNS and the matrix. As the refractive index of ionic liquid (*n* ≈ 1.4–1.6) is close to that of PSNSs, vivid colors can be hardly produced. To solve this problem, a dense thin PE layer with high transparent at IR regimes is adopted between the MWCNT film and the PSNS layer to hinder the infiltration of ionic liquid.

(4) $$W_{\text{band}}=\frac{4}{\pi }\lambda |\left(n_{ps}-n_{\text{matrix}}\right)/\left(n_{ps}+n_{\text{matrix}}\right)|$$

**Figure 2:**
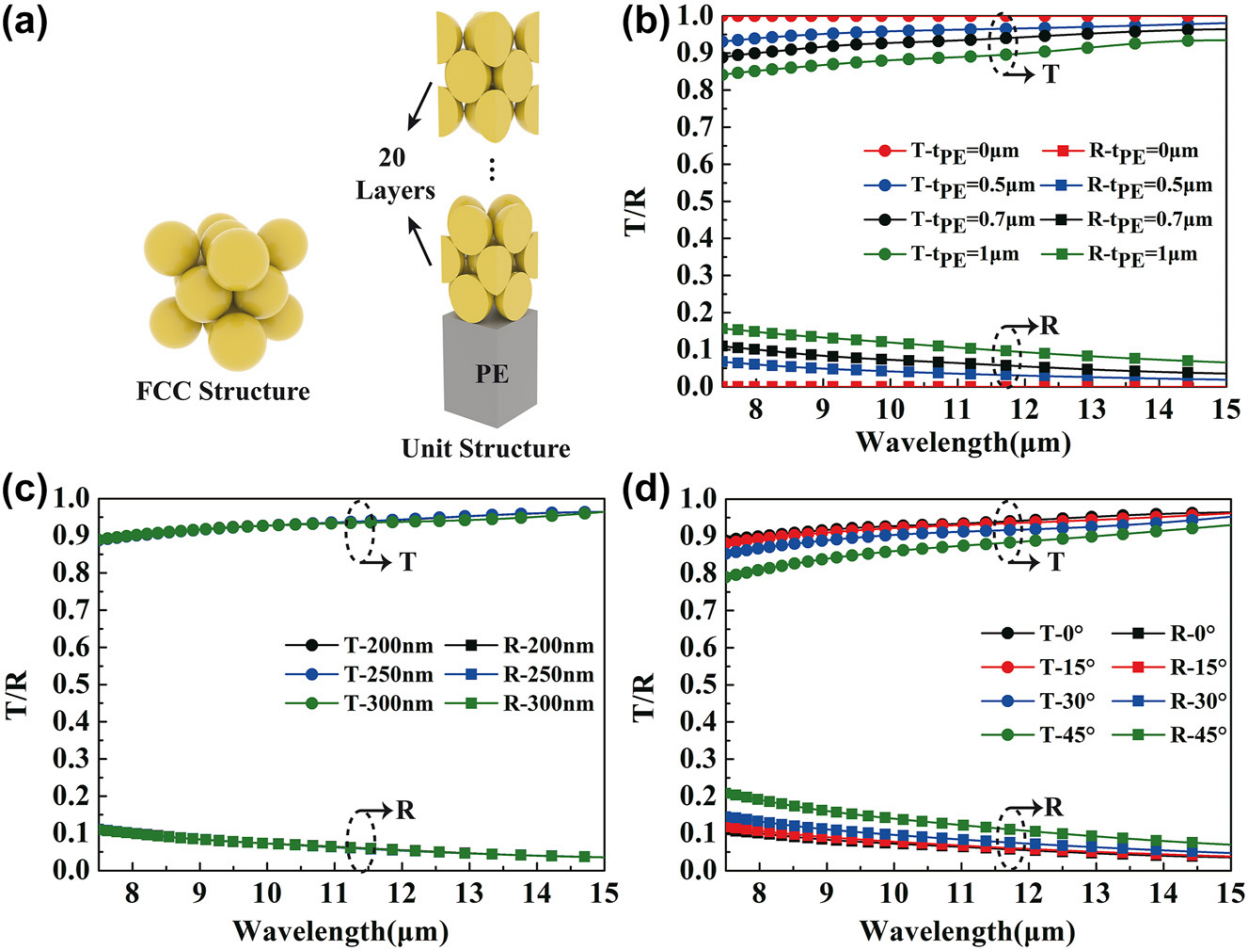
Simulation results on the upper PE/PSNS layer. (a) The simulation model with PSNSs of face centered-cubic crystal structure. (b) Simulation results of the transmission and reflection spectra for different PE thicknesses, where the PSNS diameter is set to 200 nm. (c) Simulation results of the transmission and reflection spectra for different PSNS diameters, where PE thickness is set 0.7 μm. (d) Simulation results of the angle-dependent transmission and reflection spectra, where the PE thickness and PSNS diameter are 0.7 μm and 200 nm, respectively.

In order to investigate the effect on the IR functionality, we performed numerical simulations on the transmission and reflection characteristic of the combined PE/PSNS layer, as shown in Figure 2. Here, the simulation model has the face centered-cubic crystal structure, as shown in Figure 2(a). According to existing report [5], the better thickness of the PSNS layer is within 3–4 μm, corresponding to about 20 stacking layers of the PSNS. As seen from Figure 2(b), the transmission declines slightly while the reflection enhances accordingly with the PE thickness increasing. The transmittances maintain no less than 0.93, 0.90, and 0.84 over the simulated spectral range for the 0.5, 0.7, and 1 μm-thick PE layers, respectively. Theoretically, higher IR transparency of the PE/PSNS layer is better. However, easy preparation and mechanical property should also be taken into account. In this study, the IR transparency of PE/PSNS layer exceeding 0.85 is thought to be acceptable. As seen from Figure 2(c), when the thickness of the PSNS layer is within 3–4 μm, the simulated transmission and reflection spectra almost remain unchanged for different PSNS diameters, demonstrating almost no influence of the PSNS diameter on the IR transmission/reflection characteristic. In addition, the transmission/reflection performances under oblique incidence were also taken into account. As can be seen in Figure 2(d), the transmittance of the combined PE/PSNS layer exhibits a decrease tendency with the incident angle increasing, but it can still maintain more than 0.8 even for 45-degree incidence, verifying good angle stability. In brief, the appearance color can be regulated by customizing the PSNS diameter. Due to the high IR transparency of the PSNS layer as well as PE layer, the VIS function can be easily overlaid into the MWCNT-based electrochromic IR layer, successfully realizing the VIS-IR compatible and independent camouflage with multicolor patterns and tunable emissivity.

### 2.2 Experiment and output performances

**Figure 3:**
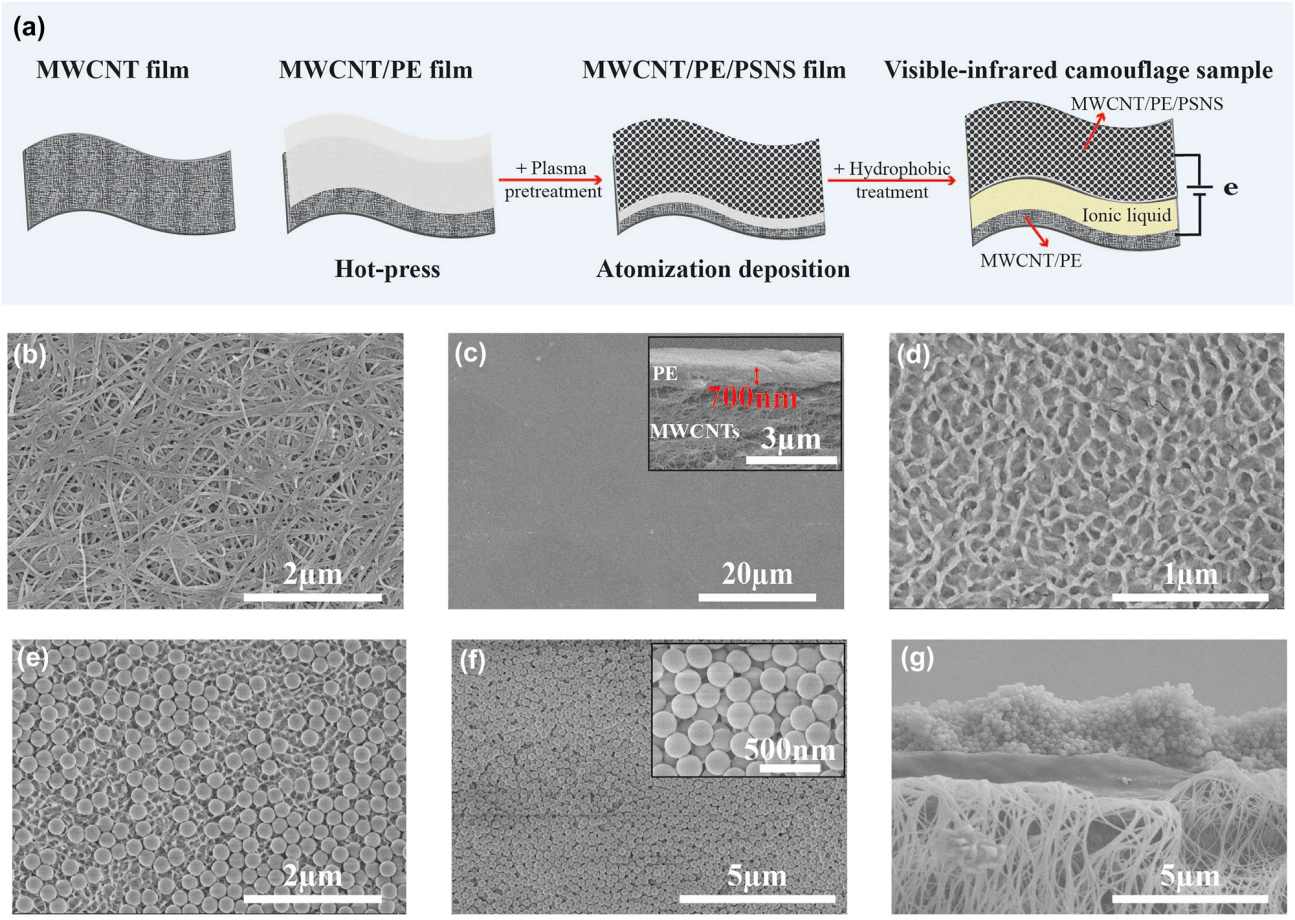
Fabrication and characterization of our VIS-IR compatible camouflage device. (a) Preparation procedure of the sandwich-structured camouflage device. (b–g) SEM images showing (b) unprocessed MWCNT film, (c) the MWCNT/PE composite film before plasma pretreatment (the insert: cross section of the film), (d) surface morphology of the PE film after plasma pretreatment, (e) PSNS distribution morphology for about 1 min atomization deposition (f) PSNS distribution morphology for about 9 min atomization deposition (the insert: zoomed image) and (g) the cross section of the MWCNT/PE/PSNS composite film.

Figure 3(a) illustrates the fabrication procedure of our camouflage device, which includes hot-pressing process of the MWCNT/PE composite film, plasma pretreatment on the PE side of the MWCNT/PE film, atomization deposition of PSNSs, hydrophobic treatment with hexamethyldisilazane (HMDS) and final device assembly (see more details in experimental section). Surface morphology and cross-section structure for critical preparation steps are also characterized by scanning electron microscope (SEM). Figure 3(b) showcases the surface morphology of unprocessed MWCNT film, from which, it can be seen that carbon nanotubes intertwining with each other to form the highly porous structure. After hot-pressing processing, MWCNTs were tightly bonded with PE film, whose surface is dense and smooth, as shown in Figure 3(c). The thickness of PE layer is about 700 nm, which can be seen from the cross-section image (see the inset). Subsequently, plasmon treatment was applied on PE surface to form shallow pit defects, as shown in Figure 3(d). These shallow pit defects can not only effectively enhance the adhesive strength between the PE film and PSNSs, but also increase the uniformity of the PSNS distribution, as presented in Figure 3(e), where the PSNS atomization deposition time lasts about 1 min. In order to obtain the required thickness of the PSNS layer, the atomization deposition time needs to last about 9 min. As seen from Figure 3(f) and (g), PSNSs are self-assembled on the PE film uniformly and compactly with the thickness of 3–4 μm, forming the isotropic short-range ordered structure.

**Figure 4:**
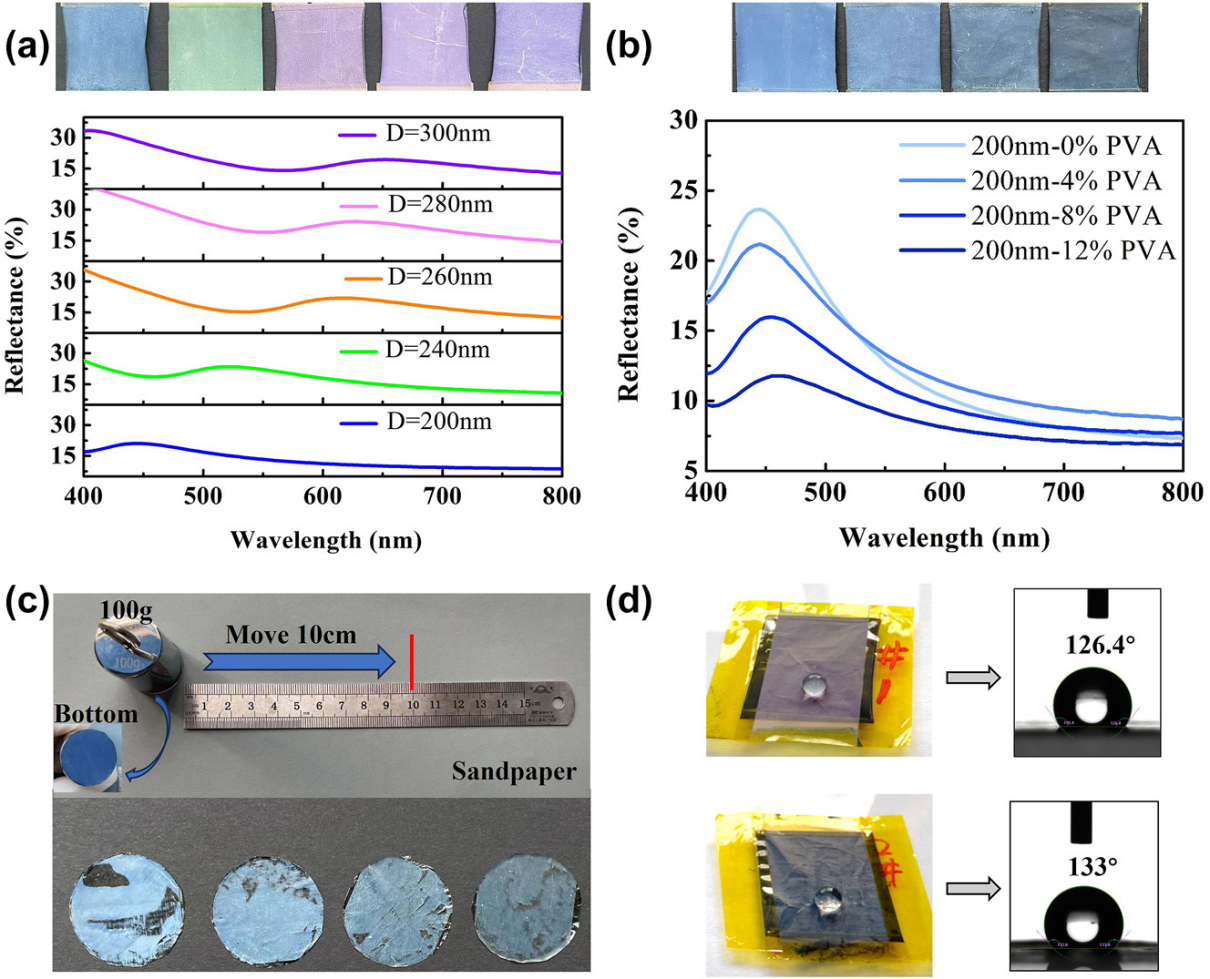
Optical properties of PSNS layer and physical characteristic of the MWCNT/PE/PSNS film. (a, b) Optical photographs along with the reflection spectra of the PSNS layer for different (a) diameters and (b) PVA concentrations, respectively. (c) Picture of the abrasion test and abrasion-resistant testing results of samples with different PVA contents, which are 0 wt%, 4 wt%, 8 wt%, and 12 wt% from left to right. (d) Experimental verification of the hydrophobic property for the purple (300 nm PSNSs) and blue (200 nm PSNSs) films.

Subsequently, we experimentally investigated the color rendering function as well as the physical characteristic for the MWCNT/PE/PSNS composite film. Through customizing the PSNS diameter, different appearance colors can be obtained. Here, a color system including blue, green and purple is obtained as the PSNS size changes from 200 to 300 nm, as shown in Figure 4(a). As can be seen, the characteristic wavelengths of the reflection spectra are located at 445 nm, 520 nm, 615 nm, 630 nm, and 650 nm for the PSNS diameters of 200 nm, 240 nm, 260 nm, 280 nm, and 300 nm, respectively. According to Equations (1)–(3), When *n*_*ps*_ ≈ 1.6, *n*_matrix_ ≈ 1.0 (air) and *φ*_*ps*_ ≈ 0.74, the stopband positions (*λ*) are calculated as 478 nm, 573 nm, 621 nm, 669 nm, and 717 nm for D = 200 nm, 240 nm, 260 nm, 280 nm, and 300 nm, respectively. In above calculations, *φ*_*ps*_ (≈0.74) approaches the PSNS volume fraction of ideal FCC crystal structure. Actually, it is difficult to well stack PS microspheres together to form ideal FCC crystal structure in practical deposition process, meaning *φ*_*ps*_ is not precise 0.74. Meanwhile, there is a deviation in the size of the purchased PS microspheres. Thus, the deviation between the measurement and calculation results is attributed to irregularly stacking structure and the particle size deviation in reality. It is also worthy pointing out that pure yellow or red colors are not produced because of the resonant Mie scattering and backscattering resonance of individual PSNSs [5], [41], [42]. In order to improve the adhesion of the PSNS layer, polyvinyl alcohol (PVA) was added as the bonding agent, which could increase the interface strength not only among PSNS individuals but also between the PSNS and the PE film. However, the added PVA amount also has effect on the appearance color. Herein, four different MWCNT/PE/PSNS composite films with PVA concentrations of 0 wt%, 4 wt%, 8 wt%, and 12 wt% were prepared by the atomization deposition with the same duration time of 9 min, where the PSNS diameter was 200 nm, as shown in Figure 4(b). It can be observed the film brightness decreases with the increasing PVA concentration, which is also well demonstrated by the measured reflection spectra. This phenomenon can be explained as the reduction of the refractive index contrast between the PSNS and the air matrix after incorporating the PVA (*n* ≈ 1.48). Even so, the PVA concentration almost has no influence on the characteristic wavelength of the reflection spectrum. We also performed the abrasion-resistant testing for the prepared MWCNT/PE/PSNS composite film, which was stuck at the bottom of a standard 100 g weight and moved on sandpaper for the 10 cm distance within 2 s, as shown in Figure 4(c). According to the abrasion-resistant testing results, it can be seen that the damage degree of the film surface is significantly relieved with the PVA concentration increasing. Therefore, the adding amount of PVA should be taken into account for balancing the brightness against the mechanical strength. Besides, we also treated the device surface with hexamethyldisilazane (HMDS). Due to the nanoscale morphology generated by PSNSs and low surface energy of HMDS, good hydrophobic performance can be realized. As seen from Figure 4(d), the water contact angles reach 126.4° and 133° for purple (300 nm PSNSs) and blue (200 nm PSNSs) films, respectively.

**Figure 5:**
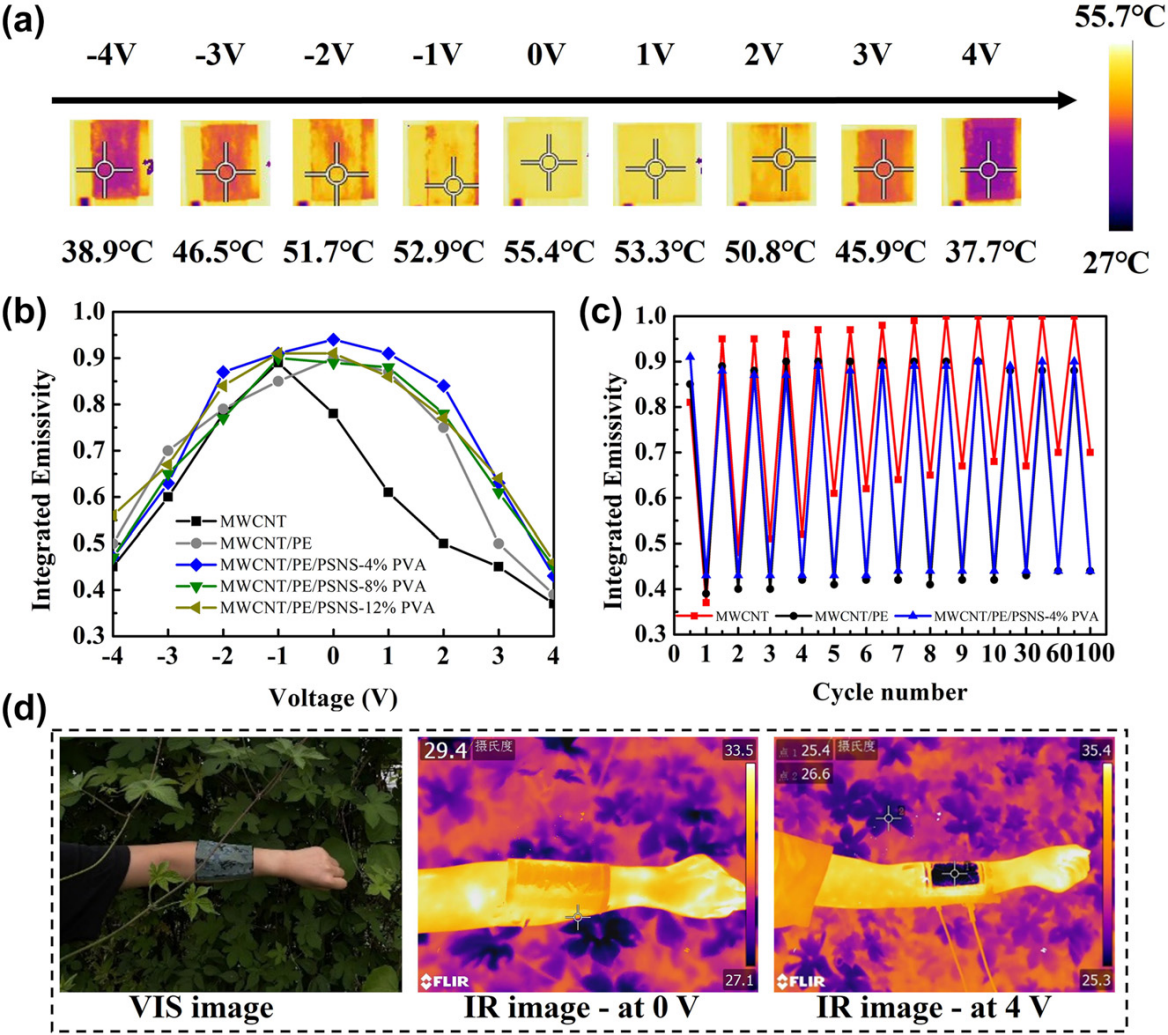
The IR camouflage performance of the VIS-IR compatible sample with the size of 2.5 cm × 2.5 cm. (a) Thermal images for different bias voltages varying from −4 V to 4 V. (b) The integrated emissivity as a function of the bias voltage for different upper films. (c) Cycling measurement results of the integrated emissivity for different upper films. (d) VIS and IR images of our devices covered on the arm.

After verifying the VIS performance, we further investigated the dynamic IR function of our device. Here, a blue-colored prototype was placed on a thermostat stage with the surface temperature of 70 °C. As the biasing voltage changed from -4 V to 4 V, corresponding thermal images were recorded by an IR camera, as shown in Figure 5(a). It can be seen that the apparent temperature decreases with the absolute biasing voltage increasing, e.g., as the biasing voltage varies from 0 to 4 V, the prototype realizes the suppression of 17.7 °C (i.e., from 55.4 °C to 37.7 °C). In addition, the color of the whole device didn’t change when infrared emissivity varied by electrical bias (see the measurement results of the reflection spectra of a blue device under different biasing voltages in Figure S1). Meanwhile, we also compared the IR emission performances of the devices for different upper electrode films. They are unprocessed MWCNT film, MWCNT/PE film, MWCNT/PE/PSNS with 4 wt% PVA film, MWCNT/PE/PSNS with 8 wt% PVA film, and MWCNT/PE/PSNS with 12 wt% PVA film, respectively. Figure 5(b) plots the obtained integrated emissivity as a function of the biasing voltage for each case. As can be seen, for the case where the unprocessed MWCNT film is used as the upper electrode, the integrated emissivity presents the maximum value at the biasing voltage of about -1.0 V accompanying with a nearly linear change tendency at both sides; for other cases, the integrated emissivity exhibits the change tendency like a downward parabola, showing tiny distinction under the same biasing voltage. This phenomenon may be attributed to the difference of the complicated movement of free ions for presence and absence of the PE film, which needs to be further explored. In particular, the maximum IR emissivity of device with unprocessed MWCNT film as upper electrode is -1.0 V. This is because the initial MWCNT usually exhibits the p-type characteristic due to oxygen impurities at the atmosphere [25], [26], [43]. As we known the highest infrared emissivity of carbon nanotubes is observed at the neutral state. Once negative gating voltage is applied, cations will be driven to the MWCNT and accumulated on its surface, producing n-type doping. Reversely, p-type doping occurs under applying the positive gating voltage. Hence, the unprocessed p-type MWCNT film could return to the neutral state through proper n-type doping, i.e., applying some specified negative gating voltage (-1.0 V). Whereas, MWCNT/PE film in our device was processed by the high-temperature heat treatment (see the MWCNT/PE film fabrication in experimental section), which can effectively reduce defects in the MWCNT. Hence, the maximum integrated emissivity in cases of processed MWCNT/PE film occurs at 0 V. According to the collected results, when applying the biasing voltage of 4 V, the integrated emissivity values are 0.37, 0.39, 0.43, 0.45, and 0.46 for devices with unprocessed MWCNT film, MWCNT/PE film, MWCNT/PE/PSNS with 4 wt% PVA film, MWCNT/PE/PSNS with 8 wt% PVA film and MWCNT/PE/PSNS with 12 wt% PVA film, respectively. It is noted that the integrated emissivity only increases by 0.02 (i.e., from 0.37 to 0.39) for introducing the PE film, experimentally demonstrating the thin PE film has excellent IR transparency; by contrast, after depositing PS/PVA mixture onto the MWCNT/PE films, the increment of the integrated emissivity gradually grows from 0.04 to 0.07 as the PVA concentration changes from 4 wt% to 12 wt%. Considering the optical and mechanical performances, our VIS-IR camouflage device adopts the MWCNT/PE/PSNS with 4 wt% PVA as the upper electrode. Furthermore, we also made the comparison on the cycling stability for devices with unprocessed MWCNT film, MWCNT/PE film and MWCNT/PE/PSNS with 4 wt% PVA as upper electrode films. In the measurement, the testing device was still placed on a thermostat stage with the surface temperature of 70 °C, and the biasing voltage was cyclically changed between two end values corresponding to the maximum and minimum integrated emissivity. As seen from Figure 5(c), after applying 100 cycling bias, the modulation range of the integrated emissivity degenerates from 0.43 (0.37–0.8) to 0.3 (0.7–1) for the unprocessed MWCNT case, while it changes from 0.46 (0.39–0.85) to 0.44 (0.44–0.88) and from 0.47 (0.43–0.9) to 0.46 (0.44–0.9) for the MWCNT/PE and MWCNT/PE/PSNS cases, respectively. Hence, it can be concluded the PE film can help to improve the cyclic stability of the device. Lastly, a 10 cm × 10 cm device with grass camouflage pattern was cover on human arm to demonstrate its VIS-IR compatible camouflage performance. And the photograph of the grass camouflage sample shows clear division of different color patterns (see Figure S2). As seen from Figure 5(d), the arm skin which is covered with the device matches well with the bush background, realizing low visual recognition; On the contrary, the bare skin clearly stands out even with the naked eyes. In addition to the camouflage effect in the visible region, the device has infrared camouflage capability against the thermal imaging camera as well. Because the human body temperature is usually higher than that of the surrounding environment, when the device is inactive, the covered area along with bare skin clearly stands out from the thermal image; however, when applying the voltage of 4 V to the device, the thermal temperature of the covered area comes to approach that of the background, showing good IR camouflage performance. It is worthy pointing out that the visual appearance of the device maintains unchanged during altering the biasing voltage. Hence, our design can realize the visible-infrared compatible and independent camouflage.

We also compared our device with some reported VIS-IR compatible camouflage structures from four aspects, i.e., external stimulus, visible performance (multicolor pattern), infrared performance (emissivity/temperature modulation), independence of functionalities, as listed in Table 1. It can be seen that our device exhibits comprehensive advantage for advanced VIS-IR compatible camouflage.

**Table 1:** Comparison with reported VIS-IR camouflage materials.

Structure	External stimulus	Multicolor pattern	Infrared emissivity/temperature modulation	Independence of functionalities	Ref.
ITO/NiOx/LiTaO_3_/WO_3_/ITO	Electrical	X	0.37 (0.49–0.86, 8–14 μm) 0.30 (0.46–0.76, 2.5–25 μm)	–	[20]
MLG/IL/Gold	Electrical	X	0.5 (0.3–0.8, 2.5–25 μm)	–	[29]
MWCNT/IL/MWCNT	Electrical	X	0.55 (0.15–0.7, 7.5–13 μm)	–	[25]
MLG/IL/Li-NMC-coated Al foil thermochromic	Electrical	X	0.45 (0.25–0.7, 2–18 μm)	X	[27]
Layer/thermoelectric layer	Thermal	√	40 °C (10 °C–50 °C)	X	[19]
Au-MXene/Fe_3_O_4_@polyaniline	Thermal and mechanical	X	0.8 (0.1–0.9, 2.5–25 μm)	X	[37]
ITO/NiO/Ta_2_O_5_/Li/WO_3_/ITO/Ge/HfO_2_	Electrical	√	0.2 (2.5–25 μm)	√	[22]
PSNS/PE/MWCNT/IL/MWCNT/PE/	Electrical	√	0.47 (0.43–0.9, 7.5–13 μm)	√	**This work**

## 3 Conclusions

In summary, we have demonstrated a VIS-IR compatible camouflage, which can independently realize color-patterned VIS appearance and tunable IR emissivity. The VIS camouflage function is implemented by upper PSNS coating. To realize the large-area color-patterned preparation, atomization depositing is adopted in this study. The lower electrochromic IR layer takes advantage of MWCNTs as the electrode as well as the IR active material. Experimental results of a proof-of-concept prototype (with a size of 10 cm × 10 cm) reveal that different colors (including blue, green and purple) have been obtained, and the IR emissivity can be electrically regulated from 0.43 to 0.9. Moreover, the prototype shows almost no degradation of the VIS-IR camouflage performance after 100 charge/discharge cycles. Besides, the water contact angle of the outmost surface exceeds 120°, implying good hydrophobic characteristic. Hence, this report paves an effective approach to promote multispectral compatible materials, which may find significant applications in camouflage fields as well as energy conservation fields.

## 4 Experimental section

### 4.1 Simulating calculation

The transmission and reflection spectra for the upper PE/PSNS layer were simulated by commercial software CST Microwave Studio. The unit cell condition was used in the *x*- and *y*-directions, and the open (add space) boundary was employed in the *z*-direction. The *x*-polarized planar electromagnetic wave with the incident angle changing from 0° to 45° by a step of 15° was adopted to investigate the angle dependence of the transmission and reflection. Optical constants of materials used in the simulations were modeled by fitting optical data of PE [44] and PS [45], [46], as shown in Figure S3.

### 4.2 MWCNT/PE film fabrication

The MWCNT film with the thickness of 10 μm and the electrical conductivity of 1 × 10^5^ s/m were purchased from Suzhou Tanfeng graphene Co., LTD. The porous PE membrane with the thickness of 16 μm was used to prepare the dense PE layers for isolating the penetration of ionic liquid. The preparation method of MWCNT/PE composite films was first overlapping MWCNT film and porous PE films on a vacuum absorption platform to keep flat. Then transferring the adhered films into two flat glass plates to clamp them tightly, finally putting them onto a hot plat with 180 °C for 30 min. After cooling to room temperature, the MWCNT/PE composite films were steamed off the glass plates for latter experiments.

### 4.3 MWCNT/PE/PSNS film fabrication

PSNS solution with different diameters (200 nm, 240 nm, 260 nm, 280 nm, 300 nm) were purchased from shanghai Zeyuan biological technology Co., LTD. And the concentration of PSNSs in deionized water was 2.5 wt%. In order to improve the adhesion strength, different amounts of PVA were added into the PSNSs solution with 4 wt%, 8 wt%, and 12 wt%, respectively. Concretely, the mass ratio of PVA is calculated by *W*_1_/(*W*_0_ + *W*_1_), where *W*_0_ and *W*_1_ are the weights of PSNSs and the PVA in the mixed depositing liquid. A vibrating mesh nebulizer (PN100, Medisana) with 3 µm mesh pores was used to produce aerosol. Before atomization depositing, plasma pretreated was employed to increase the surface energy of PE film for the uniform distribution of PSNSs. The PE layers were treated by plasma at 420 W for 30 s with distance of 15 cm. The fabrication process of multi-colored camouflage patterns is conducted as follows: firstly, plastic mask plates with corresponding shapes were employed to cover the intrinsic black patterned areas, and then prepared the green patterns through atomization deposition of PSNSs with the diameter of 240 nm; after the green coating solidification, these mask plates were stripped, and then the entire surface except for the blue patterned areas was covered by another plastic mask; subsequently, blue patterns at the exposed areas were prepared through atomization deposition of PSNSs with the diameter of 200 nm; after the blue coating solidification, the latter plastic mask was stripped again to finish the multi-colored patterns (see Figure S4). Finally, hydrophobic treatment was adopted on the outmost surface of MWCNT/PE/PSNS film via fumigation method using HMDS reagent.

### 4.4 The VIS-IR compatible camouflage device fabrication

The stealth device is composed of three parts. The top electrode is hydrophobic MWCNT/PE/PSNS composite film, porous PE membrane with thickness of 16 μm was placed in the middle as the spacer and the bottom electrode is MWCNT/PE composite film. DEME [TFSI] (N(2-methoxyethyl)-N-methyl-N, N-diethyl-N-ammonium bis (trifluoromethylsulfonyl)imide) as the ionic liquid was infiltrated into porous PE membrane. Both the MWCNTs side of these two electrodes were face to PE membranes, and copper conductive adhesives were attached to these two electrodes on MWCNTs side forming two wires. Finally, those films were stacked in order, and then a rubber scraper was used to remove the air bubbles between the layers.

### 4.5 Measurement

The microstructure and morphology of the PSNS layer and composite films was characterized by scanning electron microscope (SU8010, Hitachi). The abrasion test was employed to evaluate the stability of PSNS coatings with different content of PVA. Samples were stuck at the bottom of a standard 100 g weight and moved on a 3000-grit SiC sandpaper for the 10 cm distance within 2 s. The device surface wettability was test by a contact angle analyzer. The total hemispherical directional reflection in range 300–800 nm was measured by spectrophotometer (Lambda 1050, PerkinElmer). Thermal IR images were observed via FLIR T650sc with a spectral range of 7.5–13.0 μm. As we known, the IR camera measures the emitted thermal power and converts it to a temperature with a user set emissivity. Therefore, the emitted thermal power for a given IR image is fixed; the actual emissivity value can be obtained by matching the apparent temperature to the real temperature (which is measured by the thermocouple). In our experiments, the thermocouple was directly attached onto the outmost surface of the device (see Figure S5).

## Supplementary Material

Supplementary Material Details
