# Supplementary material for: Visible-infrared compatible and independent camouflage with multicolor patterns and tunable emissivity
Source: Nanophotonics. 2024 May 17;13(17):3123–33. doi: 10.1515/nanoph-2024-0125 (PMC11502113; doi:10.1515/nanoph-2024-0125)
Supplement: Supplementary file 1 — Supplementary Material Details [file j_nanoph-2024-0125_suppl_001.docx]

**Supporting Information**

**Visible-infrared compatible and independent camouflage with multicolor patterns and tunable emissivity**

*Yuetang Wang, Liming Yuan, Yong Mao, Cheng Huang^*^, Jingkai Huang, Xiaoliang Ma, Yuzhuo Qi, Yang Liu, He Lin and Xiangang Luo^*^*


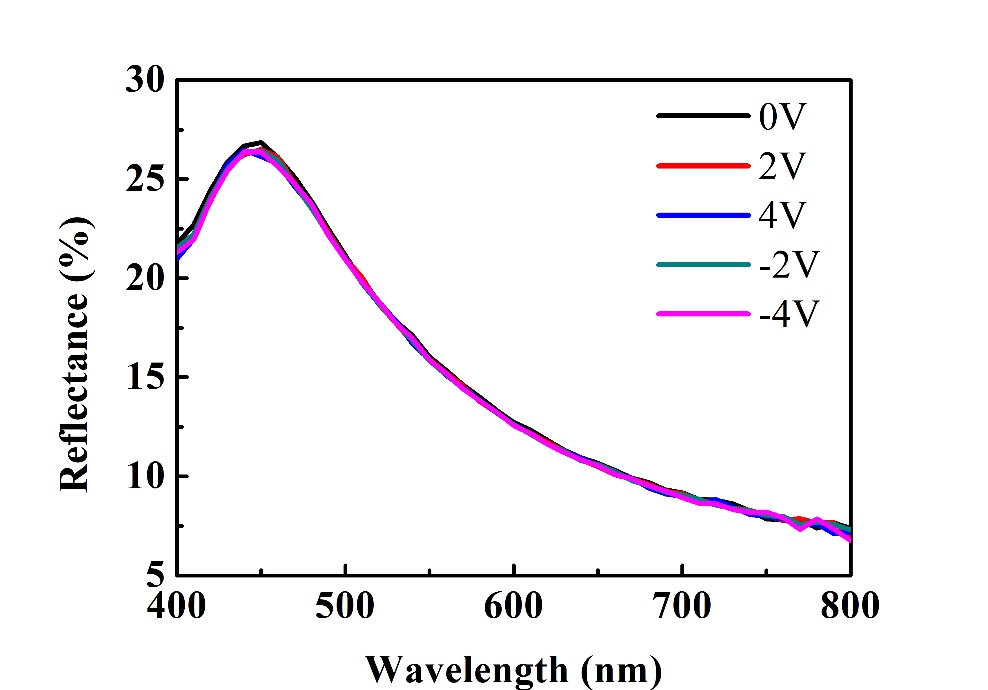


**Figure S1:** Measured reflection spectra of a blue device under different biasing voltages.


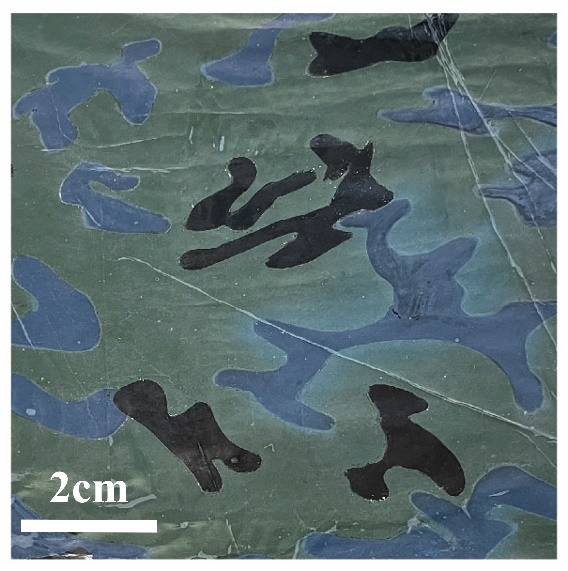


**Figure S2:** Photograph of grass camouflage pattern sample.


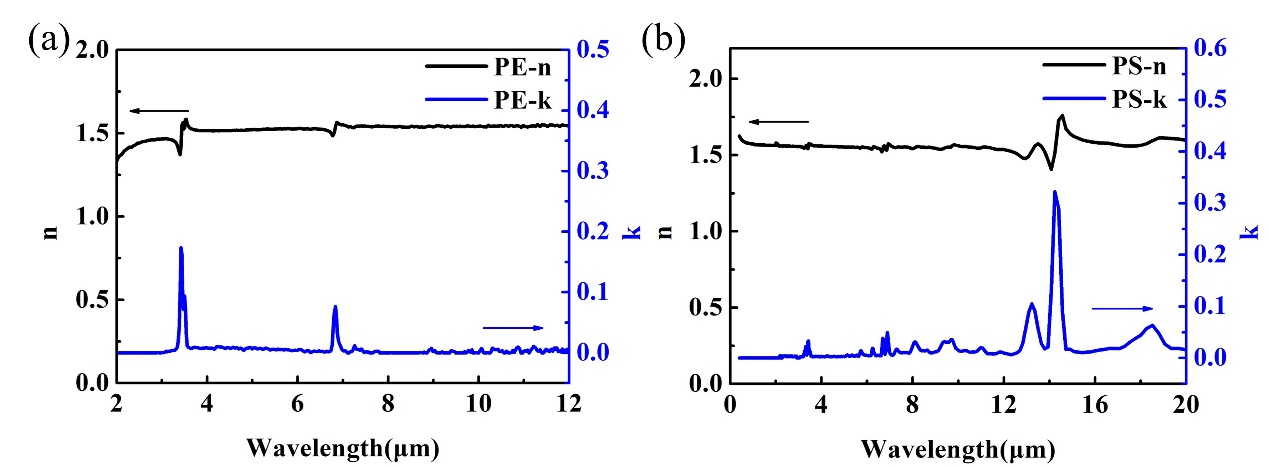


**Figure S3:** Complex refractive index of PE (a) and PS (b).


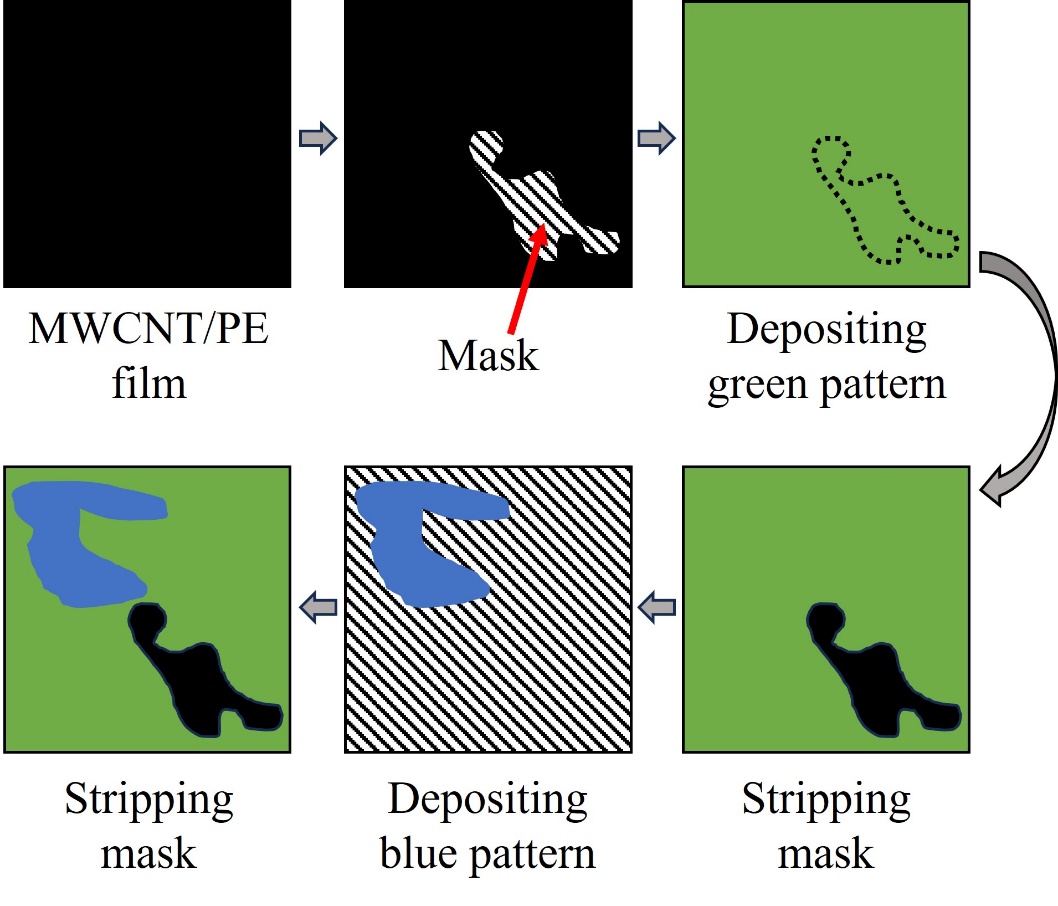


**Figure S4:** Fabrication process diagram of multi-colored camouflage patterns.


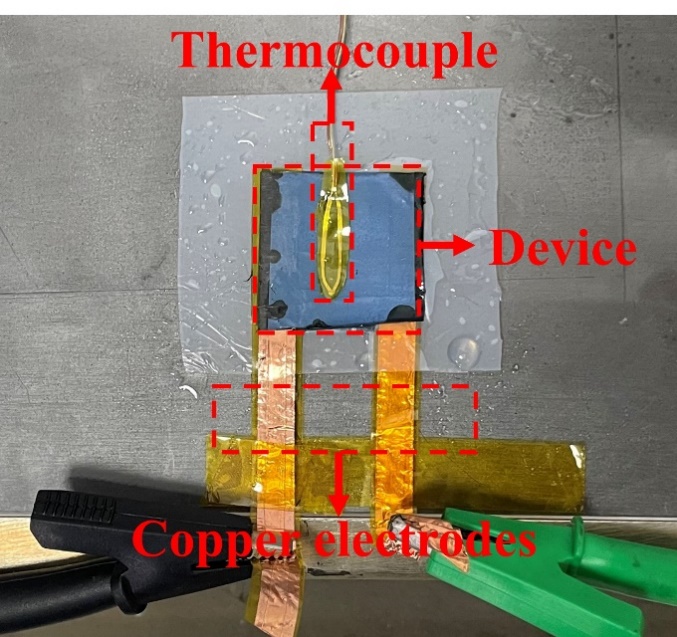


**Figure S5:** Photograph of the measurement setup of the sample surface temperature by a thermocouple.
